# Supplementary material for: Interaction of Arsenic Exposure and Transcriptomic Profile in Basal Cell Carcinoma
Source: Cancers (Basel). 2022 Nov 15;14(22):5598. doi: 10.3390/cancers14225598 (PMC9688807; doi:10.3390/cancers14225598)
Supplement: Supplementary file 1 [file cancers-14-05598-s001.zip › Form_S1_Skin Biopsy Pathology Report.pdf]

Bangladesh Vitamin E and Selenium Trial (BEST)
Skin Biopsy Pathology Report

BEST-017 **Version 3 – 18 May 2009**

Specimen ID: ☐☐☐☐☐☐ **Date reported:** __ __ / __ __ / __ __ __ __ (d d / m m / y y y y)

NO.	FEATURE	CODING CATEGORY			
1a	Parakeratosis	1=Yes	2=No		
1b	If yes, parakeratosis	1=Focal	2=Diffuse		
2	Acanthoses	1=Yes	2=No	3=Artifactual	
**SCC SCORE**					
3a	Hyperchromasia	1=Yes (1 pt)	2=No		**Note:**<br>0 pt: Other<br>1-2 pts: Mild dysplasia<br>3-4 pts: Moderate dysplasia<br>5-6 pts: Severe dysplasia<br>7 pts: Bowen's<br>8 pts: Invasive SCC
3b	Nuclear enlargement	1=Yes (1 pt)	2=No		
3c	Pleiomorphism	1=Yes (1 pt)	2=No		
3d	Dermal invasion	1=Yes (1 pt)	2=No		
3e	If at least one yes in 3a-3d, then affected:	1=Lower 1/3 only (1 pt)<br>2=Lower 2/3 only (2 pts)<br>3=Upper 3/3 but not full thickness (3 pts)<br>4=Full thickness (4 pts)			
**BCC SCORE**					
4a	Basaloid	1=Yes (1 pt)	2=No		**Note:**<br>0-2 pt: BCC r/o (Other)<br>3-4 pts: Step sections needed<br>5-7 pts: BCC
4b	Mitosis	1=Yes (1 pt)	2=No		
4c	Apoptosis	1=Yes (1 pt)	2=No		
4d	Palisading nuclei	1=Yes (1 pt)	2=No		
4e	Stromal retraction	1=Yes (1 pt)	2=No		
4f	Myxoid stroma	1=Yes (1 pt)	2=No		
4g	Invasion	1=Yes (1 pt)	2=No		
**MICROSCOPIC DIAGNOSIS**					
5	Microscopic diagnosis	1=Arsenical keratosis →			***Dysplasia:***<br>1=Absent<br>2=Mild<br>3=Moderate<br>4=Severe
		2=In-situ squamous cell ca (Bowen's)			
		3=Invasive squamous cell ca →			***Differentiation:***<br>1=Well<br>2=Moderate<br>3=Poor
		4=Basal cell ca →			***Type:***<br>1=Superficial<br>2=Nodular
		5=Other (Specify) →			

Pathologist: ______________________________
